# Differentiation of cerebrospinal fluid inflammatory biomarkers between neonatal post-hemorrhagic and congenital hydrocephalus

**DOI:** 10.1186/2045-8118-12-S1-P15

**Published:** 2015-09-18

**Authors:** Gakwaya Habiyaremye, Diego M Morales, Clint D Morgan, James P McAllister, David D Limbrick

**Affiliations:** 1 Department of Neurological Surgery, Division of Pediatric Neurosurgery, Washington University, St. Louis, MO, USA; 2 Vanderbilt University School of Medicine, Nashville, TN, USA

## Introduction

Neonatal Post-hemorrhagic hydrocephalus (PHH) develops partly due to an inflammatory process occurring after intraventricular hemorrhage whereas the majority of congenital hydrocephalus (CH) results from primary and secondary CNS malformations. We hypothesize that cerebrospinal fluid (CSF) content of inflammatory biomarkers is higher in neonatal PHH relative to CH. To test this hypothesis, we measured CSF concentrations of CCL-3, CXCL-12, CX3CL-1, IL-10 and P-selectin in both conditions.

## Methods

ELISA was used to measure CSF inflammatory biomarker concentrations in 10-15 patients per study group. Study groups included PHH-LP (lumbar puncture), congenital hydrocephalus (CH), and pre-term controls (PT). PHH-LP and PT samples were collected perinatally during spinal tap; CH samples were collected ventricularly at time of reservoir implantation.

## Results

CCL-3 was significantly increased in PHH relative to both CH and PT (PHH-LP>CH with p= 0.0002; PHH-LP>PT, p=0.0001). P-selectin was significantly elevated in PHH compared to CH and PT (PHH-LP>CH, p=0.0002; PHH-LP>PT, p=0.0009). IL-10 was significantly elevated in PHH-LP relative to PT (PHH-LP>PT, p=0.0001). No significant differences were found in CXCL-12 and CX3CL-1. Study group comparison did not show any difference between CH and PT.

## Conclusions

Our findings suggest that PHH may be distinguished from CH based on its higher levels of CCL-3 and P-selectin. However, CX3CL-1, CXCL-12 and IL-10 may not be useful in distinguishing both conditions. Interestingly, it appears that CH may not be distinguished from PT controls based on levels of inflammatory biomarkers. These findings confirm our hypothesis that inflammatory biomarkers are higher in neonatal PHH and indicate that the use of inflammatory modulators could be more beneficial to PPH versus CH.

